# Development and evaluation of a one-step multiplex real-time TaqMan^®^ RT-qPCR assay for the detection and genotyping of equine G3 and G14 rotaviruses in fecal samples

**DOI:** 10.1186/s12985-019-1149-1

**Published:** 2019-04-25

**Authors:** Mariano Carossino, Maria E. Barrandeguy, Erdal Erol, Yanqiu Li, Udeni B. R. Balasuriya

**Affiliations:** 1 Louisiana Animal Disease Diagnostic Laboratory and Department of Pathobiological Sciences, School of Veterinary Medicine, Louisiana State University, Baton Rouge, LA USA; 2 Escuela de Veterinaria, Universidad del Salvador, Champagnat 1599, Ruta Panamericana km54.5 (B1630AHU), Pilar, Buenos Aires, Argentina; 3 Instituto de Virología, CICVyA, INTA. Las Cabañas y Los Reseros s/n, (1712) Castelar, Buenos Aires, Argentina; 4 Department of Veterinary Science, University of Kentucky Veterinary Diagnostic Laboratory, University of Kentucky, Lexington, KY USA; 5 Maxwell H. Gluck Equine Research Center, Department of Veterinary Science, University of Kentucky, Lexington, KY USA

**Keywords:** Rotavirus A, Equine rotavirus A, ERVA, One-step multiplex RT-qPCR, G-typing, G3, G14, Foal diarrhea

## Abstract

**Background:**

Equine rotavirus A (ERVA) is the leading cause of diarrhea in neonatal foals and has a negative impact on equine breeding enterprises worldwide. Among ERVA strains infecting foals, the genotypes G3P[12] and G14P[12] are the most prevalent, while infections by strains with other genomic arrangements are infrequent. The identification of circulating strains of ERVA is critical for diagnostic and surveillance purposes, as well as to understand their molecular epidemiology. Current genotyping methods available for ERVA and rotaviruses affecting other animal species rely on Sanger sequencing and are significantly time-consuming, costly and labor intensive. Here, we developed the first one-step multiplex TaqMan^®^ real-time reverse transcription polymerase chain reaction (RT-qPCR) assay targeting the NSP3 and VP7 genes of ERVA G3 and G14 genotypes for the rapid detection and G-typing directly from fecal specimens.

**Methods:**

A one-step multiplex TaqMan^®^ RT-qPCR assay targeting the NSP3 and VP7 genes of ERVA G3 and G14 genotypes was designed. The analytical sensitivity was assessed using serial dilutions of in vitro transcribed RNA containing the target sequences while the analytical specificity was determined using RNA and DNA derived from a panel of group A rotaviruses along with other equine viruses and bacteria. The clinical performance of this multiplex assay was evaluated using a panel of 177 fecal samples and compared to a VP7-specific standard RT-PCR assay and Sanger sequencing. Limits of detection (LOD), sensitivity, specificity, and agreement were determined.

**Results:**

The multiplex G3 and G14 VP7 assays demonstrated high specificity and efficiency, with perfect linearity. A 100-fold difference in their analytical sensitivity was observed when compared to the singleplex assays; however, this difference did not have an impact on the clinical performance. Clinical performance of the multiplex RT-qPCR assay demonstrated that this assay had a high sensitivity/specificity for every target (100% for NSP3, > 90% for G3 VP7 and > 99% for G14 VP7, respectively) and high overall agreement (> 98%) compared to conventional RT-PCR and sequencing.

**Conclusions:**

This new multiplex RT-qPCR assay constitutes a useful, very reliable tool that could significantly aid in the rapid detection and G-typing of ERVA strains circulating in the field.

## Background

Equine rotavirus A (ERVA) has been identified as the leading cause of diarrhea in neonatal foals < 3 months of age and is responsible for 20 to 77% of foal diarrhea cases, causing significant economic losses to the equine breeding enterprises [1–7]. Rotaviruses are icosahedral, non-enveloped viruses with a double-stranded, segmented RNA genome (dsRNA) that belong to the family *Reoviridae* (genus *Rotavirus*) [8, 9]. The ERVA genome consists of 11 double-stranded RNA segments that encode for six structural proteins (VP1–4, 6 and 7) and six non-structural proteins (NSP1–6). Segment 11 encodes for two non-structural proteins (NSP5 and NSP6) [1, 10]. The rotavirus particle consists of a triple capsid, including an outer capsid composed of VP7 and VP4, an intermediate layer integrated by VP6 and an inner capsid formed by VP1, VP2 and VP3 [11–13]. The two outer capsid proteins, VP7 and VP4, are the most variable and immunogenic proteins of the virus, which independently elicit neutralizing antibodies following infection [1, 14]. Based on VP6 identity, rotaviruses are classified into eight groups (A-H), from which group A rotaviruses (RVA) are the leading cause of diarrhea in humans and several animal species, including horses [15]. Group A rotaviruses are further classified into G-types and P-types according to the nucleotide sequence of the two outer capsid proteins, VP7 and VP4, encoded by segments 9 and 4 of the genome, respectively [16]. Currently, 27 G-types and 35 P-types of RVA have been recognized in several species including humans [9]. Thus far, seven G-types (G3, G5, G6, G8, G10, G13 and G14) and six P-types (P[1], P[3], P[7], P[11], P[12] and P[18]) have been identified among the RVA affecting horses, with G3P[12] and G14P[12] being the most prevalent and epidemiologically relevant genotypes [1, 2, 17–19]. Other genomic arrangements involving G- and P-types different from G3/G14 and P[12] have been infrequently described as infecting horses [1].

Group A rotaviruses are transmitted through the fecal-oral route and infection in young foals is associated with life-threatening watery diarrhea induced by a combination of malabsorptive, osmotic and secretory mechanisms [1, 20]. Control of ERVA infection in young foals is achieved by the routine vaccination of pregnant mares with an inactivated vaccine and strict husbandry/hygienic practices to reduce the viral burden in the environment [1, 7, 21–23]. ERVA vaccines have been shown to aid in the reduction of the incidence and severity of diarrhea and also in the intensity and duration of viral shedding, however they do not guarantee full protection [1, 21, 22]. In addition, previous studies have shown that there is significant antigenic variation among ERVA genotypes, which leads to emergence of viruses that are not neutralized by antibodies elicited by the current vaccines [24–29]. Moreover, temporal and spatial variations in the prevalence and distribution of ERVA genotypes has been previously reported [2, 29, 30]. Therefore, it is important to perform genotypic characterization of ERVA strains in order to understand the molecular epidemiology of ERVA, identify novel viral reassortants and potential interspecies transmission, and assess vaccine performance in the field. Currently, sequencing of VP7, VP4 and other genome segments are required for genotyping circulating rotavirus strains. Conventional sequencing methodologies are generally labor intensive, low throughput and costly. Real-time reverse transcription quantitative polymerase chain reaction (RT-qPCR) assays, particularly TaqMan^®^ assays, offer a wide spectrum of advantages compared to conventional RT-PCR and sequencing. Some of these advantages include high throughput sample processing, increased sensitivity and specificity, faster turnaround time, and ability to multiplex. Even though several singleplex and multiplex RT-qPCR assays have been developed for the genotyping of human RVA genotypes [31–35], none have been developed for the genotyping of animal rotaviruses thus far, including ERVA. Here, we developed and evaluated the performance of a one-step multiplex RT-qPCR assay that allows the rapid detection of ERVA and the genotyping of the most frequent G-types affecting horses (G3 and G14) in fecal specimens. Overall, the one-step multiplex RT-qPCR assay developed in this study can simultaneously detect and genotype G3 and G14 ERVA strains with a performance equivalent to that of conventional VP7-specific RT-PCR and Sanger sequencing.

## Methods

### Cell lines and viruses

MA-104 cells (ATCC^®^ CRL-2378.1™, American Type Culture Collection [ATCC], Manassas, VA, USA) were maintained in Eagle’s minimum essential medium (EMEM, Cellgro^®^, Mediatech Inc., Herndon, VA, USA) with 200 mM L-glutamine and 10% inactivated fetal bovine serum (Atlanta Biologicals, Flowery Branch, GA, USA), 1 mM sodium pyruvate, 1X non-essential amino acids, penicillin and streptomycin (100 U/ml and 100 μg/ml) and 0.25 μg/ml of amphotericin B (Gibco^®^, Carlsbad, CA, USA). Tissue culture fluid (TCF) derived from MA-104 cells infected with ERVA strain H2 (G3P[12]), ERVA strains RVA/Horse-tc/ARG/E8701-5MCCH/2016/G14P[12], RVA/Horse-tc/ARG/E8701–6MCBI/2016/G14P[12] and RVA/Horse-tc/ARG/E8701-9MCGR/2016/G14P[12]; bovine RVA (BRVA) strain NCDV-Lincoln, BRVA strain B223 and simian RVA strain SA11 were used to assess the RT-qPCR assay’s performance and specificity. Briefly, confluent monolayers of MA-104 cells were inoculated with a 1:10 dilution of TCF containing trypsin-activated RVA in a minimal volume of maintenance media without FBS. After 1 h adsorption at 37 °C, monolayers were overlaid with MA-104 medium containing 0.5 μg/ml of trypsin type IX (Sigma-Aldrich, St. Louis, MO) and without FBS, and incubated at 37 °C and 5% CO_2_ until 100% cytopathic effect was observed (48 h post infection). Infected flasks were frozen/thawed, clarified by centrifugation at 1,500 *X* g for 15 min at 4 °C, aliquoted, and stored at − 80 °C.

### Viral RNA and bacterial DNA

RNA and DNA from the following viruses and bacteria associated with diarrhea in horses were included for specificity evaluation of the ERVA-specific RT-qPCR assay: TCF containing ERVA strains RVA/Horse-tc/GBR/H2/1976/G3P[12], RVA/Horse-tc/ARG/E8701-5MCCH/2016/G14P[12], RVA/Horse-tc/ARG/E8701–6MCBI/2016/G14P[12] and RVA/Horse-tc/ARG/E8701-9MCGR/2016/G14P[12] [29]; TCF containing bovine RVA (BRVA) strains RVA/Cow/United States/NCDV-Lincoln/1969/G6P6[1] and RVA/Cow/United States/B223/1983/G10P8[11], TCF containing simian RVA strain RVA/Simian-tc/ZAF/SA11-N5/1958/G3P[2], TCF containing equine coronavirus strain NC99 [36], and TCF containing equine rhinitis A (NVSL-0600EDV8501) and B (NVSL-0610EDV85010) viruses. ERVA strain H2, BRVA strains NCDV-Lincoln and B223, and simian RVA strain SA11 were kindly provided by Dr. Viviana Parreño (INTA, Buenos Aires, Argentina). Equine rhinitis viruses were obtained from the National Veterinary Services Laboratories, United States Department of Agriculture, Ames IA. DNA samples from *Escherichia coli, Salmonella enterica, Rhodococcus equi, Neorickettsia risticii, Clostridium perfringens, Clostridium difficile* and *Lawsonia* *intracellularis* were obtained from the University of Kentucky Veterinary Diagnostic Laboratory (Table 1).

**Table 1:** A panel of viruses and bacteria associated with diarrhea in horses, cattle and simians was used to assess the specificity of the singleplex and multiplex RT-qPCR assays for detection and genotyping of ERVA

Viruses	Bacteria
ERVA strain RVA/Horse-tc/GBR/H2/1976/G3P[12]	*Escherichia coli*
ERVA strain RVA/Horse-tc/ARG/E8701-5MCCH/2016/G14P[12]	*Salmonella enterica*
ERVA strain RVA/Horse-tc/ARG/E8701–6MCBI/2016/G14P[12]	*Rhodococcus equi*
ERVA strain RVA/Horse-tc/ARG/E8701-9MCGR/2016/G14P[12]	*Neorickettsia risticii*
BRVA strain RVA/Cow/United States/NCDV-Lincoln/1969/G6P6[1]	*Clostridium perfringens*
BRVA strain RVA/Cow/United States/B223/1983/G10P8[11]	*Clostridium difficile*
SRVA strain RVA/Simian-tc/ZAF/SA11-N5/1958/G3P[2]	*Lawsonia intracellularis*
ECoV strain NC99	
ERAV strain NVSL-0600EDV8501	
ERBV strain NVSL-0610EDV85010	

ERVA, equine rotavirus A; BRVA, bovine rotavirus A; SRVA, simian rotavirus A; ECoV, equine coronavirus; ERAV, equine rhinitis A virus; ERBV, equine rhinitis B virus

### Fecal samples

A total of 177 fecal samples from diarrheic foals were used in this study. Among these, 112 fecal samples were collected from farms in central Kentucky [29] while 65 were from outbreaks of diarrhea that occurred in Argentina between 2009 and 2014 [29, 30]. Ten percent fecal suspensions in serum-free EMEM were prepared, centrifuged at 2500 *X g* for 15 min at 4 °C, then filtered through a 0.45 μm syringe filter. Aliquots of fecal suspensions were stored at − 80 °C.

### Nucleic acid isolation

Nucleic acid isolation was performed using the taco™ mini nucleic acid extraction system (GeneReach USA, Lexington, MA, USA) as previously described [37]. Two hundred microliters of 10% fecal suspension or tissue culture supernatant was used as sample input and elution was performed with 200 μl of elution buffer and stored at − 80 °C for future use.

### RT-PCR amplification of ERVA VP7 gene (segment 9)

We established a VP7-specific (gene segment 9) standard RT-PCR assay using the Qiagen One-Step RT-PCR kit (Qiagen, Valencia, CA, USA) as previously described [38]. This assay was used as the gold-standard method for ERVA detection in fecal specimens [2, 39]. Briefly, a 25 μl reaction mixture was composed of 5 μl 5X One-Step RT-PCR Buffer, 1 μl dNTP Mix, 1 μl of VP7-specific forward and reverse primers (Table 2, 20 μM, final concentration 0.8 μM), 1 μl of One-Step RT-PCR Enzyme Mix, 11 μl of RNase-free water and 5 μl of template previously subjected to a denaturing step at 95 °C for 5 min. The cycling conditions included a reverse transcription step (50 °C for 30 min) followed by a PCR activation step at 95 °C for 15 min; 35 cycles of denaturation (94 °C for 1 min), annealing (47 °C for 1 min) and extension (72 °C for 2 min); and a final extension at 72 °C for 2 min. PCR amplification products yielded a 1062 bp band following electrophoretic separation in a 1% agarose gel.

**Table 2:** Primers used for RT-PCR amplification and sequencing of VP7 (genome segment 9) of ERVA

Primer name	Target	Nucleotide Position	Sequence (5′ to 3′)	Application
RVAVP7-Gra-5	VP7	1-20^a^	GGCTTTAAAAGCGAGAATTT	RT-PCR and sequencing
RVAVP7-Gra-3	VP7	1062–1,044^a^	GGTCACATCATACAACTCT	RT-PCR and sequencing
RVAVP7–389-R	VP7	389-370^a^	CCAGTAGGCCATCCTTTAGT	Sequencing
RVAVP7-635-F	VP7	635-659^a^	GTCCACTTAATACACAAACTCTAGG	Sequencing
RVAVP7-241-R	VP7	245-220^a^	GCAGTRTCCATTGAACCAGTAATTG	Sequencing
RVAVP7-852-F	VP7	856-879^a^	GAYATAACGGCTGATCCAACTACG	Sequencing
RVAVP7-881-F	VP7	885-906^a^	CTCCACAGATTGGACGAATGA	Sequencing

^a^nucleotide position based on GenBank Accession number KM454508.1

### Sequencing of ERVA VP7 gene for G-typing

Sequencing of the full-length VP7 gene (genome segment 9) was performed using a high fidelity One-Step RT-PCR kit (Qiagen One-Step *Ahead* RT-PCR kit) and the forward and reverse primers RVAVP7-Gra-5 and RVAVP7-Gra-3 (Table 2) as previously described [29]. Briefly, a 25 μl reaction mixture was composed of 10 μl 2.5X One-Step *Ahead* RT-PCR Master Mix, 1 μl of VP7-specific forward and reverse primers (20 μM, final concentration 0.8 μM), 1 μl of 25X One-Step *Ahead* RT-Mix, 7 μl of RNase-free water and 5 μl of template previously subjected to a denaturing step at 95 °C for 5 min. The cycling conditions included a reverse transcription step (45 °C for 15 min) followed by a PCR activation step at 95 °C for 5 min; 40 cycles of denaturation (95 °C for 15 s), annealing (47 °C for 15 s) and extension (68 °C for 2 min); and a final extension at 68 °C for 5 min. PCR products (1062 bp) were gel-purified using the QIAquick^®^ Gel Extraction kit (Qiagen) according to the manufacturer’s recommendations. DNA was submitted for Sanger sequencing to a commercial company (Eurofins Genomics LLC, Louisville, KY, USA). Both DNA strands of VP7 amplicons were sequenced using a panel of primers specified in Table 2. Sequence analysis was performed using Geneious R7 (Biomatters Inc., Newark, NJ, USA). G-types were identified using an automated genotyping tool for RVA (RotaC 2.0, http://rotac.regatools.be/) [40].

### Accession numbers

The nucleotide sequences derived from the fecal samples and tissue culture fluid corresponding to ERVA strains RVA/Horse-tc/ARG/E8701-5MCCH/2016/G14P[12], RVA/Horse-tc/ARG/E8701–6MCBI/2016/G14P[12] and RVA/Horse-tc/ARG/E8701-9MCGR/2016/G14P[12] utilized in this study were deposited in GenBank under accession numbers MG970165-MG970197, MH458234-MH458237, KP116019-KP116049 and MF074190-MF074212.

### Primer and probe design

Multiple alignments of full-length ERVA G3 (*n* = 17) and G14 (*n* = 39) VP7 nucleotide sequences derived from GenBank were performed and consensus sequences obtained using Geneious R7 software. G-type specific forward and reverse primers and probes were designed towards conserved regions specific to G3 VP7 and G14 VP7 gene sequences using the PrimerQuest tool (https://www.idtdna.com/Primerquest/home/Index) (Table 3). The primer and probe sequences were checked for specificity using the NCBI Basic Local Alignment Search Tool (BLAST; https://blast.ncbi.nlm.nih.gov/Blast.cgi?PROGRAM=blastn&PAGE_TYPE=BlastSearch&LINK_LOC=blasthome) while self-annealing sites, hairpin loop formation and 3′ complementarity were verified using the IDT OligoAnalyzer tool (https://www.idtdna.com/calc/analyzer).

**Table 3:** Primers and probe combinations for the detection of rotavirus A (pan-rotavirus A, targeting the NSP3 gene) and specific amplification of the VP7 gene of equine rotavirus A G3 and G14 genotypes

Name	Target	Nucleotide Position	Sequence (5′ to 3′)
NVP3-FDeg^1^	NSP3	963-982^a^	ACCATCTWCACRTRACCCTC
NVP3-R1^1^	NSP3	1053-1,034^a^	GGTCACATAACGCCCCTATA
NVP3-Probe^1^	NSP3	984-1,026^a^	Cy5-ATGAGCACAATAGTTAAAAGCTAACACTGTCAA-BHQ2
RVA-G3-756F	VP7 (G3)^b^	756–777	GATGTTACCACGACCACTTGTA
RVA-G3-872R	VP7 (G3)^b^	872–854	AGTTGGATCGGCCGTTATG
RVA-G3-779P	VP7 (G3)^b^	779–823	FAM-TGGGACCACGAGAGAATGTAGCTGT-TAMRA
RVA-G14-ARG869F	VP7 (G14)^c^	869–885	ATCCGACTACGGCTCCA
RVA-G14-ARG1011R	VP7 (G14)^c^	1011–990	TGCAGCAGAATTTAATGATCGC
RVA-G14-ARG886P	VP7 (G14)^c^	886–915	HEX-CAGATTGGACGAATGATGCGTATAAATTGG-MGB

^1^Primers and probe name and sequences derived from Freeman et al., 2008

^a^nucleotide position based on GenBank Accession number X81436

^b^nucleotide position based on GenBank Accession number KM454497.1

^c^nucleotide position based on GenBank Accession number KM454508.1

Cy5, cyanine 5; BHQ2, black hole quencher 2; FAM, 6-carboxyfluorescein; TAMRA, tetramethylrhodamine; HEX, hexachloro-fluorescein; MGB, minor groove binder

### Synthesis of target NSP3 and VP7 genes and preparation of in vitro transcribed RNA

ERVA-specific in vitro transcribed (IVT) RNA was synthesized in order to determine the analytical sensitivity of the ERVA-specific multiplex RT-qPCR assay. For this purpose, a 493 nt insert containing the targeted regions (NSP3 [nt position 963–1053], G3 VP7 [nt position 756–872] and G14 VP7 [nt position 869–1011] derived from ERVA strain H2 (NSP3 and G3 VP7) and ERVA strain FI23 (G14 VP7) (GenBank Accession numbers KM454500.1, KM454497.1 and KM454508.1, respectively) were chemically synthesized (GeneArt™ Gene Synthesis, ThermoFisher Scientific, Regensburg, Germany) and cloned into the pGEM^®^-3Z vector (Promega, Madison, WI) downstream of the T7 promoter (pRVA_NSP3G3G14) by a commercial company. Subsequently, *E. coli* K12 DH10B™ T1R were transformed with the construct. Transformed bacteria were cultured overnight at 37 °C with shaking (270 rpm). Plasmid DNA was purified using QIAprep Spin Miniprep kit (Qiagen, Valencia, CA) following the manufacturer’s instructions and screened by restriction digestion using the unique *EcoRI*, *BamHI*, and *HindIII* restriction sites within and flanking the insert. Sequence authenticity was confirmed by Sanger sequencing using T7 and SP6 promoter-specific primers. Plasmid DNA (1 μg) was linearized using *HindIII*, purified using the High Pure PCR Product Purification kit (Roche, Indianapolis, IN) as instructed, and 0.5 μg of plasmid DNA was used for in vitro transcription of the pRVA_NSP3G3G14 insert using the Megascript^®^ T7 Transcription kit (ThermoFisher Scientific, Waltham, MA) following the manufacturer’s recommendations. Residual plasmid DNA was removed by digestion with TURBO™ DNase (ThermoFisher Scientific) for 15 min at 37 °C. The IVT RNA product was analyzed by agarose gel electrophoresis, subjected to a clean-up procedure using the MEGAclear™ Transcription Clean-Up kit (ThermoFisher Scientific), and quantified using a NanoDrop 2000 spectrophotometer (ThermoFisher Scientific). The pRVA_NSP3G3G14 IVT RNA was stored at − 80 °C until used. The number of ERVA IVT RNA molecules per microliter (copies/μl) was calculated according to the following formula:

The concentration of ERVA IVT RNA was adjusted to 10^7^ copies/μl using nuclease-free water containing 40 ng/μl of Ambion^®^ Yeast tRNA (ThermoFisher Scientific), and serially ten-fold diluted (10^7^ - 0.1 IVT RNA copies/μl) using nuclease-free water containing Ambion^®^ Yeast tRNA.

### ERVA-specific singleplex TaqMan^®^ real-time RT-PCR assays targeting G3 VP7, G14 VP7 and NSP3 genes

Primers and probes specific for ERVA G3 VP7 and G14 VP7 were designed as described above (Table 3). The reaction was set up using the QuantiTect™ Probe RT-PCR kit (Qiagen) following the manufacturer’s recommendations. Briefly, the 25 μl reaction contained 12.5 μl of 2X QuantiTect™ Probe RT-PCR Master Mix with ROX, 0.25 μl QuantiTect™ RT Mix, 200 nM TaqMan^®^ fluorogenic probe, 500 nM of each primer, and 5 μl of template RNA (previously subjected to a denaturing step at 95 °C for 5 min). Reverse transcription and amplification were carried out in an ABI 7500 Fast Real-time PCR System (Applied Biosystems^®^, Life Technologies, Grand Island, NY). The program included 30 min at 50 °C (reverse transcription step), 15 min at 95 °C (PCR initial activation step), followed by 45 cycles at 94 °C for 15 s (denaturation) and 60 °C for 1 min (combined annealing/extension). The NSP3-specific (gene segment 7; pan-rotavirus A) assay was established in the laboratory as previously described (Table 3) [41].

### ERVA-specific multiplex TaqMan^®^ real-time RT-PCR assays targeting G3 VP7, G14 VP7 and NSP3 genes

The G3 VP7, G14 VP7 and NSP3-specific assays were multiplexed for the simultaneous identification of all genotypes (pan-rotavirus A) and G-typing of ERVA. The reaction was set up using the QuantiTect™ Multiplex RT-PCR kit (Qiagen) following the manufacturer’s recommendations. Briefly, the 25 μl reaction contained 12.5 μl of 2X QuantiTect™ Multiplex RT-PCR Master Mix with ROX, 0.25 μl QuantiTect™ RT Mix, 200 nM of each TaqMan^®^ fluorogenic probe, 200 nM of each primer, and 5 μl of template RNA (denatured at 95 °C for 5 min before being added into the reaction well). Reverse transcription and amplification were carried out in an ABI 7500 Fast Real-time PCR System (Applied Biosystems^®^). The program included 20 min at 50 °C (reverse transcription step), 15 min at 95 °C (PCR initial activation step), followed by 40 cycles at 94 °C for 45 s (denaturation) and 60 °C for 75 s (combined annealing/extension).

### Statistical analysis

Standard curves were performed using IVT RNA (10^7^ to 0.1 IVT RNA copies/μl). Coefficients of determination (*R*^*2*^) were used to assess curve fitness. PCR amplification efficiencies (%) were calculated after regression analysis using the following formula: $\mathrm{E}=\left[{10}^{-\frac{1}{\mathrm{slope}}}-1\right]\times 100$. Limit of detection with 95% confidence (LOD_95%_) was determined by statistical probit analysis (a non-linear regression model) using the commercial software SPSS 14.0 (SPSS Inc., Chicago, IL, USA) for all assays with 9 replicates per dilution ranging from 10^5^ to 1 IVT RNA copies/μl. Cycle threshold (Ct) cut-off values were determined as the average Ct + 3 standard deviations of nine replicates of the endpoint dilution [42]. Precision (within-run and between-run imprecision) of the ERVA multiplex RT-qPCR assay was determined by performing 9 replicates of IVT RNA containing 10^5^, 10^4^ and 10^3^ RNA copies/μl on the same run (within-run imprecision) or three replicates of IVT RNA containing 10^5^, 10^4^ and 10^3^ RNA copies/μl tested on two different operational days. The coefficient of variation (%) was determined for each target concentration (G3 VP7, G14 VP7, and NSP3). The performance of the ERVA multiplex RT-qPCR assay was evaluated in fecal specimens and compared to the VP7-specific RT-PCR and G-typing by Sanger sequencing. Contingency tables (2 × 2) were generated to determine the sensitivity, specificity and agreement (kappa statistic) of each target within the multiplex RT-qPCR assay.

## Results

### Analysis of fecal samples by VP7-specific RT-PCR and sequencing for determination of G-types

A total of 177 fecal samples were included in the study, from which 92 samples were confirmed negative for ERVA, while 85 were positive as determined by VP7-specific standard RT-PCR [29, 30]. From the 85 ERVA-positive samples, 58 were collected in Argentina and 27 were collected from the USA (Kentucky). Among these, 41 were confirmed as G3 genotype while 44 were confirmed as G14 genotype by sequencing of the VP7 gene. Extensive genetic and phylogenetic analysis of these samples was recently published in a separate article [29].

### Analytical sensitivity and specificity of ERVA-specific singleplex and multiplex RT-qPCR assays targeting G3 VP7, G14 VP7 and NSP3 genes

#### Analytical sensitivity of ERVA-specific singleplex RT-qPCR assays

**Fig. 1:**
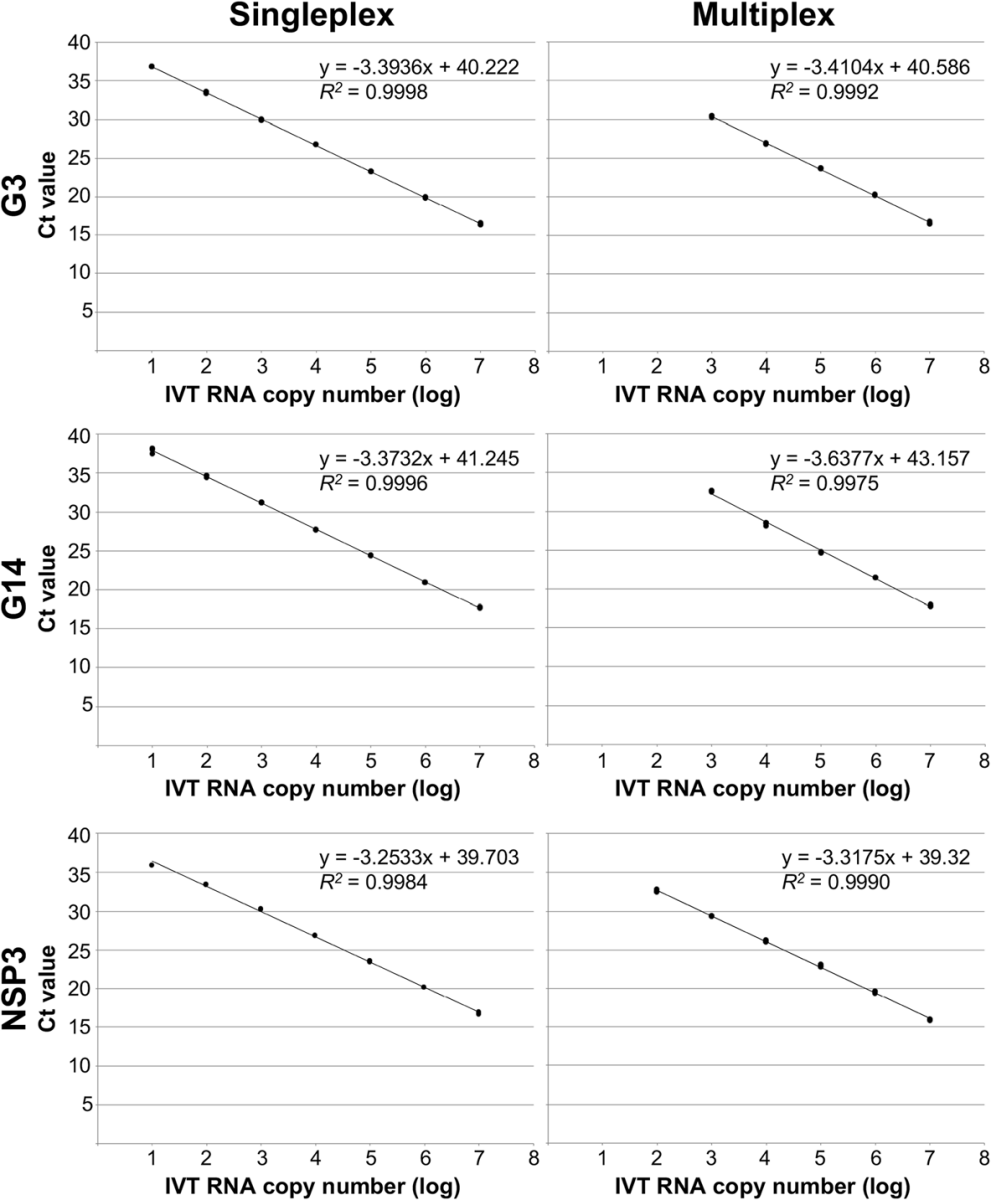
Comparison of the analytical sensitivity of the singleplex and multiplex RT-qPCR assays for the detection and G-typing of equine rotavirus A. Ct, cycle threshold; IVT RNA, in vitro transcribed RNA

The analytical sensitivity of the ERVA-specific singleplex and multiplex RT-qPCR assays was determined using a ten-fold dilution series (3 replicates per dilution) of IVT RNA (10^7^ to 0.1 IVT RNA copies/μl) containing the target sequences. Standard curves generated for the three targets (G3 VP7, G14 VP7 and NSP3) under singleplex conditions demonstrated perfect linearity (*R*^*2*^ > 0.99, Table 4 and Fig. 1). Amplification efficiencies for the G3 VP7, G14 VP7 and NSP3 targets under singleplex conditions were 97, 98% and 103%, respectively. Detection rates (100%) for the singleplex RT-qPCR assays are shown in Table 4. Probit analysis determined that the limits of detection 95% (LOD_95%_) of the G3 VP7, G14 VP7 and NSP3 RT-qPCR assays under singleplex conditions were 2.6, 5.7 and 27 copies/μl of IVT RNA and cycle threshold (Ct) cut-off points were determined at 38, 39 and 34, respectively.

**Table 4:** Analytical sensitivity analysis of singleplex and multiplex RT-qPCR assays for the detection and genotyping of equine rotavirus A

Parameter	Singleplex			Multiplex		
	G3	G14	NSP3	G3	G14	NSP3
Slope	−3.3936	−3.3732	− 3.2533	−3.4104	− 3.6377	−3.3175
Linearity (*R*^*2*^)	> 0.99	> 0.99	> 0.99	> 0.99	> 0.99	> 0.99
Efficiency (%)	97	98	103	96.4	88	100
LOD_95%_ (copies/μl)	2.6	5.7	27	716	215	47
Detection rate limit (100%, copies/μl)	10	10	100	1000	1000	100
Ct cut-off	38	39	34	32	34	34

LOD_95%_, limit of detection 95%; Ct, cycle threshold

#### Analytical sensitivity of ERVA-specific multiplex RT-qPCR assay

Standard curves generated for the three targets (G3 VP7, G14 VP7 and NSP3) under multiplex conditions also demonstrated perfect linearity (*R*^*2*^ > 0.99, Table 4 and Fig. 1). However, while the amplification efficiencies for the G3 VP7 and NSP3 targets were ± 10% of that determined under singleplex conditions (96 and 100%, respectively), a lower amplification efficiency was determined for the G14 VP7 target when multiplexing (88%). Detection rates (100%) for the multiplex RT-qPCR assay are shown in Table 4. While the 100% detection rate limit for the NSP3 assay was equal between the singleplex and multiplex formats, a 100-fold difference was observed for the G3 VP7 and G14 VP7 assays when these were multiplexed (Table 4). In comparison to the singleplex format, the LOD_95%_ were higher (716, 215 and 42 copies/μl of IVT RNA for the G3 VP7, G14 VP7 and NSP3 targets, respectively). Ct cut-off points were determined at 32, 34 and 34, respectively.

#### Analytical specificity of ERVA-specific singleplex and multiplex RT-qPCR assays

To evaluate the analytical specificity of the singleplex and multiplex RT-qPCR assays, a panel of rotavirus strains along with other viruses and bacteria associated with diarrhea in horses was used (Table 1). The ERVA-specific G3 and G14 VP7 primer-probe combinations were exclusively specific for the respective ERVA genotype, did not cross-react between each other, did not amplify other rotavirus genotypes from other species and, interestingly, did not amplify the simian SA11 strain (G3P2). The NSP3-specific primer-probe combination in both singleplex and multiplex format was specific for RVA and amplified the reference G3 and G14 strains of ERVA as well as bovine and simian rotavirus strains as previously reported [34]. None of the assays (G3 VP7, G14 VP7 and NSP3) amplified other viruses or bacteria associated with diarrhea in horses.

#### Precision assessment of the ERVA-specific multiplex RT-qPCR assay

To evaluate the precision of the multiplex RT-qPCR assay, within-run and between-run imprecision was determined as recommended [42]. In all cases, the coefficient of variation was less than 3%, indicating that the multiplex assay has a high repeatability (within-run) and reproducibility (between-run) within the range of detection (Table 5).

**Table 5:** Replication experiment to evaluate precision (within-run and between-run imprecision) of the multiplex RT-qPCR assays for the detection and genotyping of equine rotavirus A

Concentration of target (IVT RNA copies/μl)	Within-run imprecision			Between-run imprecision		
	G3	G14	NSP3	G3	G14	NSP3
100,000	2.63%	1.29%	0.89%	2.92%	1.56%	0.97%
10,000	1.75%	0.74%	0.42%	2.19%	1.24%	0.83%
1,000	2.01%	0.51%	0.56%	2.52%	0.63%	0.68%

### Clinical performance of the ERVA-specific multiplex RT-qPCR assay targeting G3 VP7, G14 VP7 and NSP3

The clinical performance of the ERVA-specific multiplex RT-qPCR assay was evaluated in a total of 177 fecal samples. The NSP3 (pan-RVA) assay was able to successfully detect ERVA in all positive samples (85/85) while no non-specific amplifications were observed in negative samples (*n* = 92; Table 6a). Therefore, the assay presented 100% sensitivity and specificity when compared to the VP7-specific standard RT-PCR assay, along with perfect agreement (kappa = 1). In the case of the G3 VP7 assay, the assay was able to correctly genotype 38/41 ERVA G3 samples while non-specific amplifications were not observed in G3-negative samples (*n* = 136, Table 6b). Only three ERVA G3 positive samples were unable to be genotyped by the multiplex assay, however these were correctly genotyped by the G3-specific singleplex RT-qPCR assay. Overall, the G3 VP7 assay presented a 92.7% sensitivity and 100% specificity when compared to the VP7-specific standard RT-PCR assay, and a high agreement (98.31% [kappa = 0.951]). Finally, the G14 VP7 assay was able to correctly identify 44/44 ERVA G14-positive samples and did not amplify 132/133 ERVA G14 negative samples (Table 6c). Consequently, the G14 VP7 assay presented a 100% sensitivity and 99.2% specificity when compared to the VP7-specific standard RT-PCR assay. The agreement between assays was high (99.44% [kappa = 0.985]). Regarding the presumed false positive sample, although this sample was determined to be an ERVA G3P[12] by Sanger sequencing, it yielded a concurrent positive amplification by the G3 and G14-specific RT-qPCR assays in both their singleplex and multiplex formats, suggesting a possible co-infection with both genotypes of ERVA.

**Table 6:** Evaluation of the clinical performance of the multiplex RT-qPCR assay for the detection and genotyping of equine rotavirus A in fecal samples compared to VP7-specific RT-PCR and sequencing (gold standard). (a) NSP3 (b) G3 VP7 and (c) G14 VP7

a				
		VP7-specific RT-PCR		
		Positive	Negative	Total
NSP3-specific RT-qPCR	Positive	85	0	85
	Negative	0	92	92
	Total	85	92	177
b				
		ERVA genotype G3^1^		
		Positive	Negative	Total
G3-specific RT-qPCR	Positive	38	0	38
	Negative	3	136	139
	Total	41	136	177
c				
		ERVA genotype G14^1^		
		Positive	Negative	Total
G14-specific RT-qPCR	Positive	44	1	45
	Negative	0	132	132
	Total	44	133	177

^1^Genotype determined by Sanger sequencing

## Discussion

Group A rotaviruses are a primary cause of diarrhea in children and animal species, including horses [1–6, 43, 44]. Even though seven G-types and six P-types of ERVA have been identified in horses, the G3P[12] and G14P[12] constitute the most epidemiologically relevant genotypes [1, 2, 17–19]. Spatial as well as temporal fluctuations between these predominant G-types (G3 and G14) of ERVA circulating in equine populations have been reported around the world [2, 30]. Interestingly, the emergent pattern of G14 ERVA and the temporal shift in the prevalent genotype has been observed in association with the implementation of widespread vaccination programs in Argentina, Japan and Ireland [2, 30, 45, 46], which rely on the use of inactivated vaccines containing only the H2 or HO-5 (G3P [12]) strains of ERVA. The difficulties faced to date in establishing cell-culture adapted G14P[12] or other strains of ERVA has precluded their inclusion into vaccine formulations. However, we have recently isolated and cell-culture adapted, three G14P[12] ERVA strains with the potential to be used as reference G14P[12] strains to study the molecular biology of this genotype and perform vaccine efficacy studies following heterologous challenge in the future [29].

In light of the antigenic differences between ERVA genotypes, their spatial and temporal distribution and their impact on vaccine efficacy, molecular surveillance and genotypification of circulating strains is critical. Since genomic arrangements of ERVA other than G3P[12] and G14P[12] are rare and the outer capsid protein VP7 contains the major neutralizing epitopes, we developed a one-step multiplex TaqMan^®^ real-time RT-PCR for the rapid detection and G-typing of the most prevalent genotypes of ERVA (G3 and G14) in fecal specimens. Compared to the conventional methods for ERVA genotyping (RT-PCR and Sanger sequencing), the multiplex RT-qPCR assay has a significantly faster turnaround time, is high-throughput, less labor-intensive and exhibits a high sensitivity, specificity and agreement as demonstrated in this study. While multiplexing did not have an impact on the detection limit of the NSP3-target, the G3 and G14 targets demonstrated a 100-fold difference in their analytical sensitivity under multiplex conditions. However, this difference in analytical sensitivity did not have a significant impact on their clinical performance on fecal specimens and only three G3 ERVA-positive samples were unable to be typed by the multiplex RT-qPCR assay (false negatives). Interestingly, these samples were correctly G-typed when the G3 VP7-specific assay was performed under singleplex conditions. Such differences are probably due to a combination of low target nucleic acid in these fecal specimens along with the 100-fold higher analytical sensitivity of the singleplex compared to the multiplex assay. Despite the low number of false negative samples (*n* = 3), all three targets (G3 VP7, G14 VP7 and NSP3) showed a high sensitivity and specificity (> 90%) along with a high level of agreement (> 98%) in the clinical specimens tested under multiplex conditions.

Noteworthy, a single sample, G-typed as G3 by means of conventional methods (RT-PCR and Sanger sequencing), exhibited specific amplification of both the G3 VP7 and G14 VP7 targets simultaneously under singleplex and multiplex conditions. Although confirmation would require RT-PCR using genotype-specific primers or next generation sequencing, due to the fact that both G3 and G14 ERVA strains were identified to be co-circulating in the same farm during the same time period, these results suggest that this dual-positive fecal specimen most likely derived from a foal that was co-infected with both G3 and G14 ERVA strains. Consequently, this may indicate that the multiplex RT-qPCR assay developed can be advantageous for the diagnosis of co-infections with G3 and G14 strains of ERVA that are currently challenging to identify. Further assessment using spiked specimens is required in order to analyze this multiplex RT-qPCR assay’s capability to identify co-infected animals. Due to the lack of reference strains and uncommon occurrence of other ERVA G-types, these were not included in this study. Therefore, it is imperative to perform Sanger sequencing on those samples that test positive for ERVA by amplification of NSP3 but are not genotyped as G3 or G14 by the current assay. In this regard, the genotyping assay developed here will facilitate rapid genotyping of circulating strains and identify rare G-types that can then be incorporated into this assay depending on their epidemiological relevance.

## Conclusions

In conclusion, the study presented herein describes the development and evaluation of a one-step multiplex TaqMan^®^ RT-qPCR assay for the detection and genotyping of the most frequent G-types of ERVA infecting horses. This assay demonstrated to have a high sensitivity, specificity and agreement compared to conventional RT-PCR and sequencing, providing rapid and reliable G-typing of ERVA strains. Therefore, this assay is highly suitable for routine diagnostics as well as to aid current surveillance programs of ERVA by rapidly characterizing circulating strains. Finally, the number of specific targets included in this assay can be updated and expanded as other genomic arrangements of ERVA emerge and become prevalent in equine populations.

## Abbreviations

BRVA: bovine rotavirus A
DNA: deoxyribonucleic acid
dNTP: deoxynucleotide triphosphate
EMEM: Eagle’s minimum essential medium
ERVA: equine rotavirus A
IVT: in vitro transcribed
LOD: limit of detection
RNA: ribonucleic acid
RT-qPCR: reverse transcription real-time polymerase chain reaction
RVA: rotavirus A
SRVA: simian rotavirus A
TCF: tissue culture fluid
